# Single subject transcriptome analysis to identify functionally signed gene set or pathway activity^a^

**Published:** 2018

**Authors:** Joanne Berghout, Qike Li, Nima Pouladi, Jianrong Li, Yves A. Lussier

**Affiliations:** Center for Biomedical Informatics and Biostatistics (CB2) & The Center for Applied Genetics and Genomic Medicine, Department of Medicine, University of Arizona, Tucson, AZ 85721, USA; CB2, Graduate Interdisciplinary Program in Statistics, University of Arizona, Tucson, AZ 85721, USA; CB2, University of Arizona, Tucson, AZ 85721, USA; CB2, University of Arizona, Tucson, AZ 85721, USA; CB2, BIO5 Institute, University of Arizona Cancer Center, Department of Medicine, University of Arizona, Tucson, AZ 85721, USA

**Keywords:** N-of-1, reproducibility, transcriptome, atherosclerosis, high fat, ontology

## Abstract

Analysis of single-subject transcriptome response data is an unmet need of precision medicine, made challenging by the high dimension, dynamic nature and difficulty in extracting meaningful signals from biological or stochastic noise. We have proposed a method for single subject analysis that uses a mixture model for transcript fold-change clustering from isogenically paired samples, followed by integration of these distributions with Gene Ontology Biological Processes (GO-BP) to reduce dimension and identify functional attributes. We then extended these methods to develop functional signing metrics for gene set process regulation by incorporating biological repressor relationships encoded in GO-BP as *negatively_regulates* edges. Results revealed reproducible and biologically meaningful signals from analysis of a single subject's response, opening the door to future transcriptomic studies where subject and resource availability are currently limiting. We used inbred mouse strains fed different diets to provide isogenic biological replicates, permitting rigorous validation of our method. We compared significant genotype-specific GO-BP term results for overlap and rank order across three replicate pairs per genotype, and cross-methods to reference standards (*limma*+FET, SAM+FET, and GSEA). All single-subject analytics findings were robust and highly reproducible (median area under the ROC curve=0.96, n=24 genotypes × 3 replicates), providing confidence and validation of this approach for analyses in single subjects. R code is available online at http://www.lussiergroup.org/publications/PathwayActivity

## 1. Introduction

While precision medicine moves towards understanding disease in individuals, transcriptome expression analysis remains largely in the realm of cohort-level understanding. In large part, this is due to the high dimension, broad range of expression values, and dynamic nature of the transcriptome. However, these qualities also mean that the transcriptome has major potential to reveal important processes during the dynamic course of a disease including onset, progression, and response to therapy^1,2^. Extracting signal from these data requires combating biological noise as well as platform and analysis pipeline factors, with variability in transcript expression levels influenced by an individual's genome sequence, environment, and stochastic processes^3-5^.

To counter these challenges, computationally focused investigations using whole transcriptome data to draw inferences about single subjects have typically required either a large set of reference samples for comparison, or used paired samples drawn from the same subject (e.g. tumor-normal) to control for a large component of individual variation. Then, they have (1) sought to find outlier expression patterns correlated with the phenotype of interest^6-9^, or (2) used clustering algorithms to bin individuals into *a priori* interpretable classifiers (ex. disease subtypes)^10,11^. Other more biologically focused approaches have limited the dimensionality of the whole transcriptome by constructing curated gene panels with known functional relevance for targeted analysis^9,12^. Combination approaches have integrated expression pattern discovery with biological knowledgebases (ex. Gene Ontology; **GO**)^13,14^ to identify functional gene set level signals that require the coordinated activation of many genes, thus reducing the dimension and impact of false positive gene-level prioritization. However, establishing how well a computational analysis method of transcript or pathway prioritization represents the ground truth of a disease is elusive and reproducibility between experiments or across analysis methods has been an issue that must be solved before transcriptomics can be applied to clinical data for non-research use, whether used for diagnosis, clinical decision making, or used to evaluate a patient's response to therapy.

We have previously described the “N-of-1-*pathways* **MixEnrich**” combination method for identification of physiologically responsive biological processes (gene sets) in a single subject using paired RNA-Seq data^15^. That study established that the MixEnrich method could recapture an average signal when applied to single subjects, though there were pathways identified by MixEnrich that did not appear as significant in the cohort analysis. In the absence of available replicate data or access to biological samples, we could not confidently establish whether these individually identified pathways were, in fact, biologically true for those patients, indicating heterogeneous characteristics relevant for personalized medicine. The goal of this study is to quantify reproducibility of single-subject results in a controlled setting with real biological data, as well to extend our bioinformatics methods development to incorporate functional logic that reveals activation, suppression or other regulatory alterations to a biological process.

Some of the most powerful genetic tools for modeling human biology are inbred laboratory mouse strains that have been maintained via brother-sister mating for over a hundred generations, becoming genetically fixed and identical (isogenic) in the process^16^. As such, within a given strain they are genotype replicates, while across strains they maintain differences and can be considered as modeling distinct individuals, exhibiting a broad range of phenotypic variation^17^. We used this principle to select a microarray dataset of inbred mice placed on an atherosclerotic high fat diet, originally published by Shockley and colleagues^18^. Biological/genotype replicates in this set allowed us to explore the data as a cohort (n=3/group) using paired *limma*^19^+FET, SAM+FET and GSEA analyses comparing high fat diet cohorts to normal diet cohorts within a genotype, or as three independent isogenic pairs by MixEnrich (3 replicate pairs of high fat:normal/strain).

## 2. Methods

### 2.1. Details of microarray and annotation datasets

We downloaded 144 raw microarray samples from GEO (GSE10493), where Shockley et al.^18^ used transcript profiling to study the effect of an atherosclerotic high fat diet on 12 inbred mouse strains. Microarray labeling errors were corrected as indicated on the Center for Genome Dynamics website (http://cgd.jax.org/datasets/expression/10strain.shtml). Briefly, male and female mice of each strain were fed a high-fat diet (30% Kcal from dairy fat) or a normal (6% fat) chow diet for 4 weeks. Then, livers were dissected and total mRNA expression profiling was done on n=3 mice per group using Affymetrix Mouse 430 v2 arrays.

Probe sets were converted to 18, 017 gene identifiers using *mouse4302mmentrezgprobe* v21, downloaded from Brainarray at the University of Michigan (http://brainarray.mbni.med.umich.edu/Brainarray/Database/CustomCDF/21.0.0/entrezg.asp). GOBiological Process (**GO-BP**) terms with gene annotations were downloaded from Mouse GenomeInformatics^20,21^ (http://www.informatics.jax.org/downloads/reports/index.html#gogene_association.mgi.gz) on 8 March 2017, and ontology terms were filtered to those 4,682 GO IDs with annotated gene set size between 15-500 (with subsumption) for increased biological resolution. The resulting GO-BP file was used for all enrichment analyses.

### 2.2. Data normalization and pre-processing

All data files were individually normalized using R/bioconductor package *SCAN.UPC* for Single-Channel Array Normalization (SCAN)^22,23^. Batch effects were removed using *ComBat*^24^ implemented via the *SVA* package^25^.

### 2.3. Creation of diet-responsive “individuals” through pairing of isogenic mice

Although we were using these data to model individual responses to diet, we could not use paired microarrays drawn from a single biological individual as collection of liver RNA is a terminal procedure, and being assigned to one diet precludes the other.

So, we created pairs of microarrays matched according to strain and sex (hereafter, “genotype”), but comprised of one chow-fed mouse and one high fat-fed mouse to create three replicates for quasi-single subject analysis. For example, if we consider the male C57BL/6J (B6) mice fed regular chow as #1-3 while those on high fat diet are #4-6, we would pair #1&4 (rep. 1), #2&5 (rep. 2), and #3&6 (rep. 3). As mice within a strain are isogenic and the experiment was conducted under rigorously controlled conditions, differences in gene expression for these pairs should represent the differences attributable to variation in the mouse's diet, plus stochastic noise. Within a strain, 0.8%^26^-11%^27^ (FDR=0.1) of transcripts expressed in mouse liver have been estimated to vary between individuals at baseline due to developmental, hormonal, circadian, and other factors. This may introduce some “false positive” results in our single-subject analyses that are more indicative of inter-individual variation than diet effect. Still, overall gene expression is highly consistent between mice of the same strain^18,28^ (Suppl. Fig. 1) and we consider this an acceptable caveat, as similar levels of variation can be observed in human blood samples collected from healthy individual volunteers over a time series^29,30^. An advantage of our model in contrast to simulations, is that by using biological replicates, we do not require manually set parameters to model noise and can capture a more realistic biological scenario – encompassing a range of expression variation, distributing that variation non-randomly across genes, and including gene-gene interactions and gene-gene expression correlation where these apply in natural systems.

### 2.4. Calculation of reference standards from cohorts (limma+FET, SAM+FET and GSEA)

To establish reference results for diet-responsive pathways in each genotype, we used three algorithmically distinct methods. For differentially expressed genes (**DEG**) followed by GO term enrichment (DEG+Enrichment) references, we used both the Linear Models for Microarray (*limma*)^19,31^ package for single channel microarray experiments and Significance Analysis of Microarrays (SAM)^32^ using *siggenes*^33^ to identify DEGs using the pairing matrix described in **2.3** and matching that used for MixEnrich analyses. For *limma*, DEGs were identified for each genotype (n=3 high fat versus n=3 normal diet) at p_adj_≤0.05 following Benjamini-Hochberg (B-H) multiple test correction. SAM was conducted as a moderated paired t-test with unequal variance with DEGs determined as significantly responsive according to within-genotype delta values based on 8 permutations and q-values calculated by the software. Deltas ranged from 0.8 (DBA/2J female) to 7.4 (CAST/EiJ female) with a median of 1.95, each selected to balance false positive and true positive rates. DEGs were used as input in a Fisher's Exact Test (**FET**) to identify overrepresented GO-BP terms at FDR_B-H_ ≤ 5% (Fig 3A).

We also created a non-parametric reference of diet-responsive GO-BP terms for each genotype using Gene Set Enrichment Analysis (GSEA)^8^ software downloaded from the Broad Institute (http://software.broadinstitute.org/cancer/software/gsea/) and implemented in Java. GSEA GO-BP terms were called significant at FDR p_adj_≤0.20, following the package developers recommended default parameters^8^. FDR was calculated via permutation after shuffling transcript labels 5000 times without replacement. Note that GSEA does not allow paired sample design.

### 2.5. Identification of individually responsive GO-BP terms using MixEnrich

We used the MixEnrich method published recently in Li *et al* (2017)^15^ to identify GO-BP terms for each sample pair, representing diet-responsive pathways in single subjects (72 files: 3 isogenic replicate pairs × 12 strains × 2 sexes). Briefly, MixEnrich uses paired data from the same subject to control for genotype effects, and models the absolute value of the log-transformed fold change (|log_2_FC|) across conditions by a probabilistic Gaussian mixture. MixEnrich assumes that these log_2_FCs follow two distributions where one corresponds to transcripts whose expression is biologically altered between the two conditions (DEGs), and the other distribution corresponds to the transcripts whose expression remains unaltered. Transcripts assigned to the “altered” distribution with a posterior probability >0.5 become inputs to an FET for identification of overrepresented GO-BP terms, with a Benjamini-Yekutieli multiple hypothesis testing correction applied (Fig. 3B). See full details and equations in Li (2017)^15^. R code for MixEnrich is available online at http://www.lussiergroup.org/publications/PathwayActivity

### 2.6. Assigning gene set functional direction in significant GO-BP terms

We mined the ontology structure of GO to identify parent-child relationships with the edge annotation of *negatively_regulates*, representing child GO-BP terms whose gene product annotations have been curated as functional repressors of the activity described in the parent (Suppl File 1)^34^. For these transcripts, an increase in expression indicates functional repression or a decrease of GO-BP activity, while a decrease in expression indicates activation of GO-BP activity, or, more accurately – removal of suppression (Fig. 3C). When transcripts with these annotations were identified as DEGs, we reversed the sign of the log_2_FC in the parent term to mirror the functional impact of that gene's change in expression on the parent term's function.

To sign a GO-BP term as activated, we identified DEGs with increased expression and no regulatory annotation (or exclusively *regulates* and/or *positively_regulates* edges), plus those genes with decreased expression and *negatively_regulates* edges in a direct child term as described. This ‘upregulated and upregulatory’ set was used in a new contingency table (Fig. 3D), and an FET for functional enrichment of BP activation was conducted. In parallel, a separate FET calculation was done to determine evidence that each GO-BP term was functionally suppressed, based on DEGs with decreased expression across the paired condition plus those with increased expression and a *negative_regulates* edge. These differ from the original FET for GO-BP term over-representation *per se* by using only the subset of signal from the (reciprocally) concordantly responsive transcripts. Final functionally signed pathway outputs were generated using the FET p-value and OR from the complete set of altered transcripts (Fig 3A), together with a categorical direction determined from the FET described in this section and Fig 3C. Functional direction was assigned as (1) activated: FDR<5% FET in up-regulated/up-regulatory and non-significant FET in down-regulated/down-regulatory, (2) reduced activity: non-significant FET in up-regulated/up-regulatory and FDR<5% in down-regulated/down-regulatory, (3) bi-directionally altered: FDR<5% in both FETs, or (4) ambiguous: non-significant in both FETs. GO-BP edge annotation files and R code are available online at http://www.lussiergroup.org/publications/PathwayActivity

## 3. Results and Discussion

### 3.1. GO-BP pathway discovery by MixEnrich, limma+FET, SAM+FET, and GSEA

Across all of the analysis methods we observed that each genotype responded differently to the high fat diet (Table 1), which was as expected based on a large body of related work including original analyses using these data^18,35-37^. GSEA tended to identify fewer pathways than limma+FET or ME, even with a more relaxed FDR threshold at 20%. This could be due to the approach's requirement that the underlying data contributing to the enrichment signal must be directionally concordant^8^. SAM+FET results were also generally low with variable numbers of significant GO-BP term results relative to the other methods for certain strains (male A/J,, B6, I/Ln, female NZB). This could be the result of the increased solution space due to use of two parameters (delta, FC), and likely represents increased stringency applied by the SAM algorithm at the chosen values. On average, 55% of MixEnrich pathways identified in a given genotype replicate pair was common to all three ME replicate analyses of the same genotype, with an average of 42% overlap between all three ME replicates and *limma*+FET, and 41% overlap with SAM+FET analysis. These values are comparable to the overlap observed between SAM+FET and *limma*+FET (41% of *limma*+FET). Exact GO-BP term overlap across all six analyses was modest, averaging 40% of the smallest input value (range: 9%-75%). Based on its ubiquitous use in transcriptome analysis, high power and robust performance with smaller sample sizes^38^, we chose *limma*+FET as our reference standard when conducting more in-depth comparisons.

### 3.2. MixEnrich identified GO-BP terms are highly convergent with reference standard

**Figure 1:**
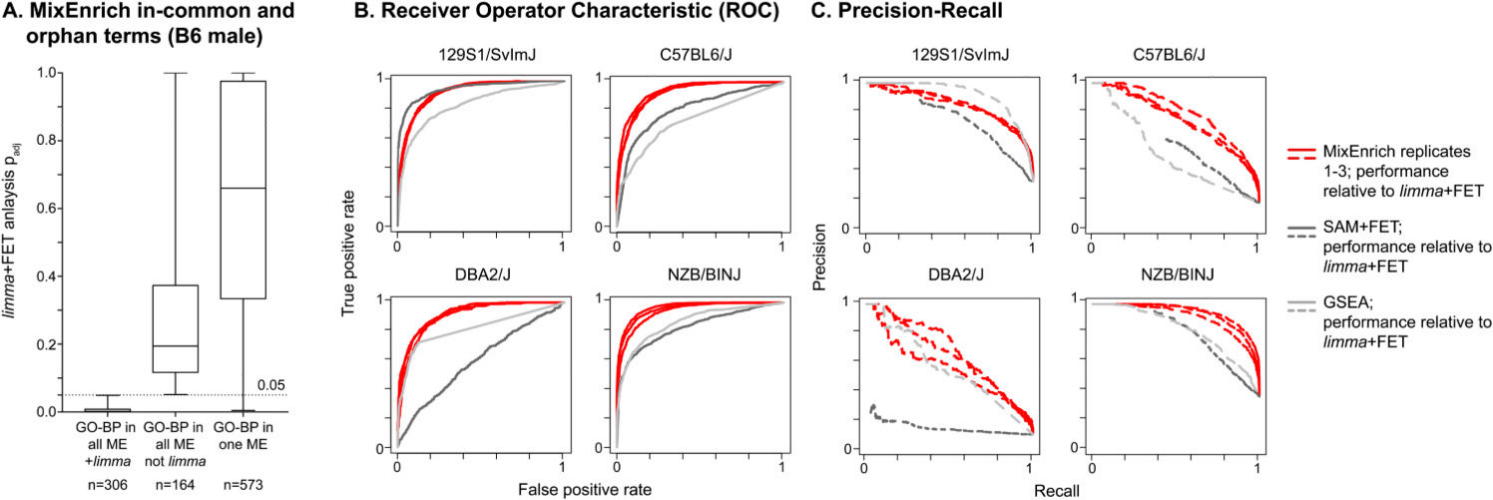
(A) Distribution of *limma*+*F*ET adjusted p-values for GO-BP terms identified in common by all 3 ME replicates in B6 male mice versus orphan GO-BPs identified by a single ME replicate. (B) ROC and (C) precision-recall curves assessing the results of GO-BP enrichment for three replicate pairs analyzed for each genotype by MixEnrich (red, three lines), or cohort-derived reference sets of n=3 high fat diet versus low fat diet mice analyzed by SAM+FET (dark gray, one line) and GSEA (light gray, one line). All curves were compared to a reference standard generated *limma*+*F*ET at FDR 5%.

We compared the set of GO-BP terms identified in-common across all three MixEnrich genotype replicates to the set of “orphan” GO-BP terms identified by only one MixEnrich replicate (Fig. 1A). Terms identified by all 3 MixEnrich replicates were highly overlapping with terms identified as significant by *limma*+FET at FDR 5%. The set of terms that were identified in common across MixEnrich replicates but not *limma* did still approach significance for the majority of cases, with a median FDR ∼20% suggesting a threshold effect. In contrast, orphan GO-BP terms were largely far from significant by *limma* analysis, achieving a median FDR ∼66%.

Next, we compared GO-BP lists using the receiver operator characteristic (ROC). ROC is most commonly used for estimating accuracy of a classifier, based on the balance between identification of true positives relative to false positives. A curve following the diagonal with area under the curve (AUC) equal to 0.5 indicates random performance, while, a curve that hugs the Y-axis up to a square turn at the top of the plot (AUC-ROC = 1) represents perfect accuracy^39^. In this case, a ‘true positive’ was defined as the MixEnrich, SAM+FET or GSEA analysis identifying a GO-BP term that was also identified by *limma+FET* analysis of that genotype (FDR 5%), and a ‘false positive’ was defined as the MixEnrich, SAM+FET or GSEA analysis calling a significant GO-BP term that the *limma+FET* analysis did not. This should be considered an approximation as the *limma* method – though widely used and well-validated – is still likely to contain errors in the totality of the result, and selecting FDR 5% as a binary decision threshold for truth is somewhat arbitrary. In addition, for these analyses, we used exact GO-BP term matching only and did not credit similar but non-identical terms appearing on the comparison lists, which is a property we hope to investigate in future analyses. Nonetheless, MixEnrich replicates were highly accurate, capturing much of the signal detected by *limma+FET* (Table 1, Fig. 1B). Across all genotypes, the median AUC-ROC for single subject MixEnrich replicates was equal to 0.96 (males: median=0.96, 1^st^ quartile= 0.94, 3^rd^ quart=0.98; females: med=0.96, 1^st^ quart=0.94, 3^rd^ quart=0.97). In contrast, SAM+FET achieved a median AUC-ROC of 0.87 (males: med=0.84, 1^st^ quart=0.80, 3^rd^ quart=0.91; females: med=0.89, 1^st^ quart=0.84, 3^rd^ quart=0.93). GSEA scored median 0.81 for males (1^st^ quart=0.67, 3^rd^ quart=0.84) and 0.69 for females (1^st^ quart=0.57, 3^rd^ quart=0.81). Fig. 1B shows curves for four representative male genotypes.

We also examined the performance of MixEnrich and GSEA in terms of precision (positive prediction value) and recall (sensitivity) (Fig. 1C). Precision and recall are related to the true positive and false positive measurements in the ROC but provide additional evaluative information in terms of relevance. Precision can be interpreted as the probability that a retrieved positive result by MixEnrich, SAM+FET or GSEA is a true positive, again, based on *limma* analysis as the reference. Recall assesses the ability of those methods to retrieve the complete list of relevant results (those matching *limma* analysis of that genotypes). We observed all three of the MixEnrich replicates outperforming both SAM+FET and GSEA for all genotypes. Relative to the other three strains, precision measurements for the DBA/2J strain were poor, likely reflecting the subtler phenotypic and liver transcriptomic responses in this strain, with accordingly short significant GO-BP pathway list in the *limma*+FET derived reference.

### 3.3. Similarity by rank based correlation

**Figure 2:**
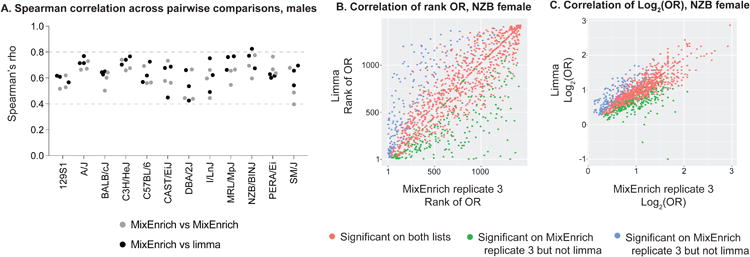
High correlation between rank order of GO-BP terms based on odds ratio. (A) high Spearman's correlation coefficient (rho) across all strains, only males shown. (B) Example plot of rank correlation using NZB female data, comparing MixEnrich replicate 3 to the limma analysis conducted across all NZB females, rho=0.75, p>10^-25^. Data are ranked from smallest to largest, so the largest ORs are ranked at (∼1500, ∼1500), rather than (1,1). (B) Example plot of log_2_(OR) values for the same pairwise comparison.

We next examined whether the GO-BP term lists were similarly ordered between the output of MixEnrich and *limma.* More than simple overlap, it is clearly important in terms of biological or clinical significance that reproducing the strongest and most salient signals should be afforded more weight than reproducing those near the bottom, as these may be of marginal significance despite meeting the set statistical threshold. We used the R package *OrderedList*^40^ to compare GO-BP rank order according to their FET-derived GO-BP odds ratios (ORs). Each MixEnrich replicate was compared to the other two and *limma*+*FET*, resulting in 6 pairwise comparisons per genotype. P-values across all strains were extremely low (p<10^-25^), supporting the assertion that the GO-BP results of MixEnrich were highly consistent across replicates, and their order was highly consistent with the GO-BP pathway order determined by *limma.* Examining a correlation plot of the ranks (Fig. 2B) further supports this, as points largely follow the diagonal with the top left corner (highest ranks, sorting from lowest OR to highest OR) highly enriched for commonality. Thus, these analyses were reproducing the same dominant signals.

We also examined each pairwise comparison using Spearman's correlation coefficient (rho; Fig. 2A) on the rank of the FET odds ratio (Fig. 2B) and log_2_OR values themselves (Fig. 2C). Correlation was high between MixEnrich genotype replicates and relative to *limma* (p-value <10^-20^ for all pairwise comparisons), with no significant difference in correlation when cross-replicates were considered (MixEnrich-to-MixEnrich) versus MixEnrich-to-*limma* comparisons (p>0.05).

### 3.4. Novel signing of GO biological process activity

**Figure 3:**
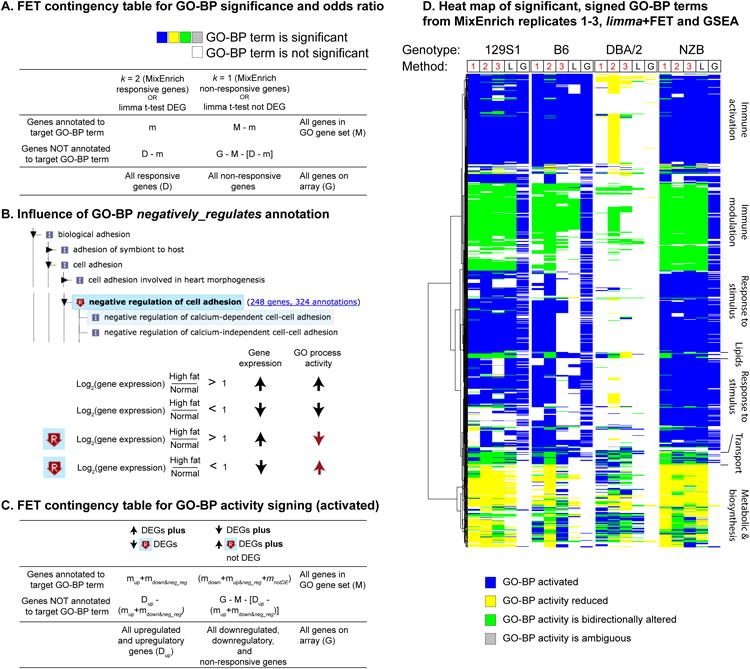
Incorporation of signed functional activation of GO-BP using *negatively_regulates*

Biologically, it is important to know whether the activity in an enriched GO-BP is increased or decreased by the disease state or intervention being studied. GSEA identifies uses concordant direction of gene expression changes, reporting results with a signed enrichment score. However, DEG+enrichment (as in *limma*+FET, SAM+FET, or MixEnrich analyses) only provides an odds ratio and statistical significance rating (p-value or FDR). Altered transcripts adding to the enrichment signal may be increased in expression, decreased, or bi-directionally split. In addition, the biological activity of genes annotated to a term can include negative regulators of that process, whose increased expression logically decreases the parent process when considered as functional biology. (Fig. 3B, 3C)^34^. In the mouse genome, 4889 unique gene products have been annotated as having negative regulatory activity in at least one biological process context (annotated to GO:0048519 or child terms), representing 20% of GO-BP annotated genes^21^. However, despite prevalence and utility, this regulatory relationship logic remains underused by the translational bioinformatics and related communities.

We incorporated ontology relationships based on *negatively_regulates* edges into our analysis as described in **Methods section 2.6** and Fig 3, then used FET on ‘upregulated and upregulatory’ genes and ‘downregulated and downregulatory’ genes separately. Comparing the results from incorporation of the *negatively_regulates* terms to a set of results without this relationship changed the categorical direction of approximately 10-15% of significant GO-BP terms, primarily shifting towards the green/bi-directionally altered category (not shown).

Plotting these significant and functionally signed GO-BP terms in a heat map (Fig. 3D) highlights several findings. First, it is again clear that the transcriptional response to high fat diet across mouse genotypes shares some common responses, and some genotype-dependent differences. In the data shown, the most notable difference is between the relatively non-responsive DBA/2J versus the other strains, though genotype-specific blocks of terms can be seen by unsupervised Euclidean clustering. Second, it is apparent that the GO-BP terms identified as significant by all of the methods and across all three biological replicates find a lot in common, as all four columns for a given genotype are visually consistent for much of their length. Third, we can see that the majority of significant DEG+enrichment identified pathways are signed in a common direction across replicates and methods. For GSEA columns (G), we used the sign of the enrichment score which results in either a unidirectional positive upregulation (blue) or negative downregulation (yellow), without the capacity for calling bidirectionality or including repressor function. As a result, the signing/color code of GSEA identified pathways appears as an outlier for a substantial number of pathways. Interestingly, GSEA also failed to identify most of the downregulated metabolic and biosynthesis pathways that comprised about 1/5 of the results in the other methods. These mechanistic responses have been verified and confirmed in other experiments^18,37^, so we consider this an omission from GSEA rather than a false positive. Looking at trends across those pathways in our data, the high fat diet appeared to increase immune and inflammatory activity, increase response to stimulus activity, and decrease metabolic biosynthesis activity, each with some GO-BP specific and genotype-specific variability.

### 3.5. Biological interpretation of results

We selected the strain comparison of NZB versus B6 to explore in greater detail. Across MixEnrich replicates and *limma*+*FET*, we found 268 common enriched GO-BP terms in response to diet with a further 190 GO-BP terms that appeared on 7/8 lists. These included many terms relating to induction of immune related responses and a reduction of cholesterol biosynthesis (Fig. 3D) which is consistent with what was reported in the original analyses of these data^18^. Comparing strain-specific responses that may underlie differential phenotypic responses, we found 16 GO-BP terms that appeared as significantly enriched in 4/4 NZB analyses and 0/4 B6 analyses, with 5 GO-BP terms significantly enriched in 4/4 B6 analyses and 0/4 NZB. NZB-specific responses were suggestive of tissue remodeling in the liver, including angiogenesis related processes, homeostasis, actin and cellular/tissue differentiation. Meanwhile, B6-specific processes were all related to ‘entry into host’, suggesting immune or cell surface receptor mediated changes.

We next determined if there were any GO-BP terms in common between the two strains where the direction of activity was opposed. Filtering the eligible list to only those GO-BP terms that were significant across all three MixEnrich replicates for both genotypes (n=436 terms), we found only one term that was significant and activated in B6 while significant and repressed in NZB mice. Cofactor catabolic process is involved in metabolism, and the participating transcripts contributing to the enrichment signal include *Acat1*, *Aldh1l1*, *Blvra*, *Blvrb*, *Cbr3*, *Hmox1*, *Hmox2*, *Ncf1*, *Nudt7*, suggesting that the specific processes of heme metabolism, Acetyl-CoA and NADPH oxidation may all be responding differentially across strains. Changes to energetic processes including each of these is consistent with what we would expect from a high fat diet, but without external validation we do not want to draw strong conclusions at transcript resolution.

## 4. Limitations and future studies

Results described here demonstrate the MixEnrich method is reproducible across isogenic replicates and provides interpretable insights at an accuracy level equivalent to other leading cross-method comparative analyses. However, there are limitations. First, GO-BP is designed to capture the normal behavior of a gene product within its cellular context^14^. If a biological process exists only within a pathogenic state (i.e. cancer), or if a gene product participates in a GO-BP exclusively in the pathogenic state, annotations will not be captured and true biology may be missed. This is a limitation of all gene set methods using GO, but should be acknowledged. In well-understood systems, a custom gene set or alternative ontology may be possible and preferred. Second, regulatory relationships may be context dependent, which we have not accounted for. Bidirectional regulatory capacity is known for certain transcription factors and epigenetic modifying proteins, depending on their interaction partners and which target(s) are being investigated. Our current strategy of treating all DEGs with *negative_regulates* edges as suppressors of their parent process may over-emphasize their down-regulatory capacity. We hope to incorporate a representation of dual annotations in the future. Third, our calculation of gene set overrepresentation via FET, though common, was not thoroughly tested, and alternative statistics (i.e. Mann-Whitney, hypergeometric) may better suit these data^41^. A final constraint on applicability of MixEnrich is that it has exclusively been tested in conditions where a paired sample approach can be used. This applies to many biological questions, but not all, and accurate interpretation of results relies on the ‘goodness’ of that assay's experimental design.

## 5. Conclusions

Developing and validating new methods capable of providing insight into the transcriptomes of individuals has the potential to provide important information in aberrant, rare, highly stratified, or clinically relevant patient-level responses. In this study we examined cross-replicate and cross-method reproducibility of GO-BP signal using the paired liver transcriptomes of isogenic mice. Overall, we the MixEnrich method was successfully reproduced the same GO-BP signals as other methods including *limma*+FET, SAM+FET and GSEA, ranking signals in roughly the same priority order as *limma*+FET. The advantage of MixEnrich is that it only requires a single sample pair and allows individualized conclusions, while the mathematics of *limma*+FET and most other comparably validated methods require at least three pairs to capture an average response.

In addition, to our knowledge, this is the first example of computational method that exploits regulatory edge relationships in signing GO-BP directionality as activated or repressed. These edges have been a component of the ontology structure since 2011^34^, but primarily used to reason with logic for knowledge representation rather than incorporated into data analysis pipelines.

## Supplementary Material

Suppl Figure 1

Suppl Table 1

## Figures and Tables

**Table 1:** Count of GO-BP terms identified for each genotype by *limma*+*F*ET (n=3 paired subjects/genotype; FDR 5%), SAM+FET (n=3 paired subjects/genotype; FDR 5%), GSEA (n=3/diet/genotype; FDR 20%), or as 3 replicate isogenic single-subjects via MixEnrich (ME; n=1 pair/genotype; FDR 5%).

	Method	129	A/J	BALB	C3H	C57BL	CAST	DBA/2	I/Ln	MRL	NZB	PERA	SM/J
Males	*limma*+FET	1034	1085	609	926	393	507	84	298	253	1317	1275	955
	SAM+FET	827	13	408	295	11	229	38	74	62	397	192	555
	GSEA	1171	1102	251	648	1181	524	19	588	21	1316	714	1203
	ME rep 1	1015	1155	934	872	865	*727*	269	816	530	1493	885	1182
	ME rep 2	1231	1121	649	752	1351	691	541	343	285	1050	868	817
	ME rep 3	970	1005	774	921	633	728	295	1170	394	1280	962	1045

	all 3 ME	670	752	439	554	470	427	166	276	199	857	580	578

	3 ME+*limma*	582	698	368	524	306	322	65	204	165	823	551	534

	all methods	368	5	67	159	1	127	6	15	14	283	123	277

Female	*limma*+FET	70	1422	156	342	123	1100	48	797	127	1165	515	140
	SAM+FET	40	171	102	93	71	99	91	245	73	119	134	88
	GSEA	150	811	0	62	31	752	12	230	39	1117	25	77
	ME rep 1	400	1521	603	474	440	843	355	652	575	932	719	241
	ME rep 2	465	1256	275	435	*252*	1235	306	528	306	1096	708	281
	ME rep 3	386	1496	361	324	450	870	273	850	318	1225	470	338

	all 3 ME	217	923	189	217	178	630	158	366	171	737	305	157

	3ME+*limma*	53	866	118	198	85	571	41	329	104	705	236	110

	all methods	5	127	0	12	3	64	9	59	9	62	6	9
