# Supplementary material for: Single subject transcriptome analysis to identify functionally signed gene set or pathway activity
Source: Pac Symp Biocomput. Author manuscript; Available in PMC 2018 Jan 1. (PMC5730358)
Supplement: Suppl Figure 1 [file NIHMS912068-supplement-Suppl_Figure_1.pdf]

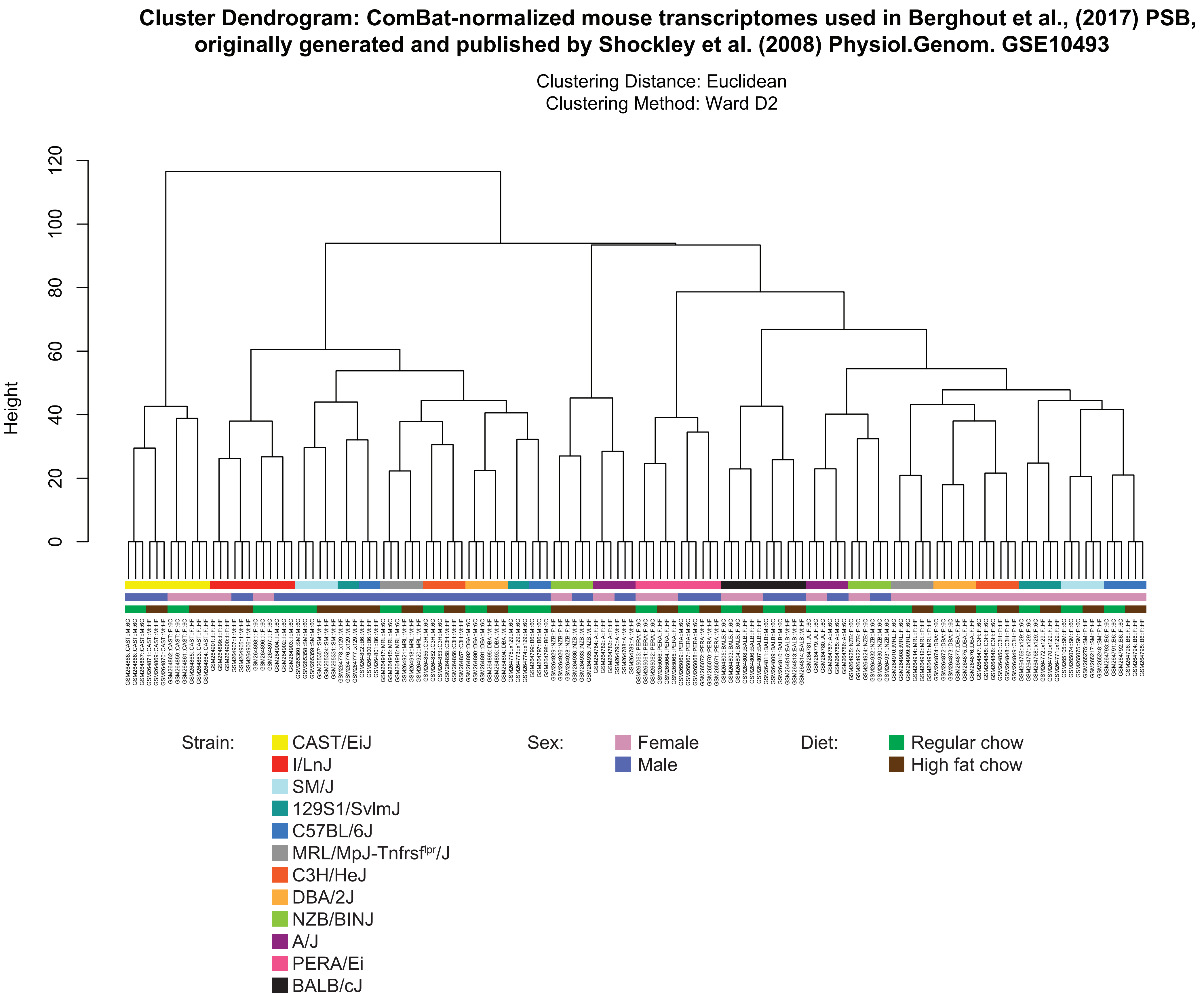

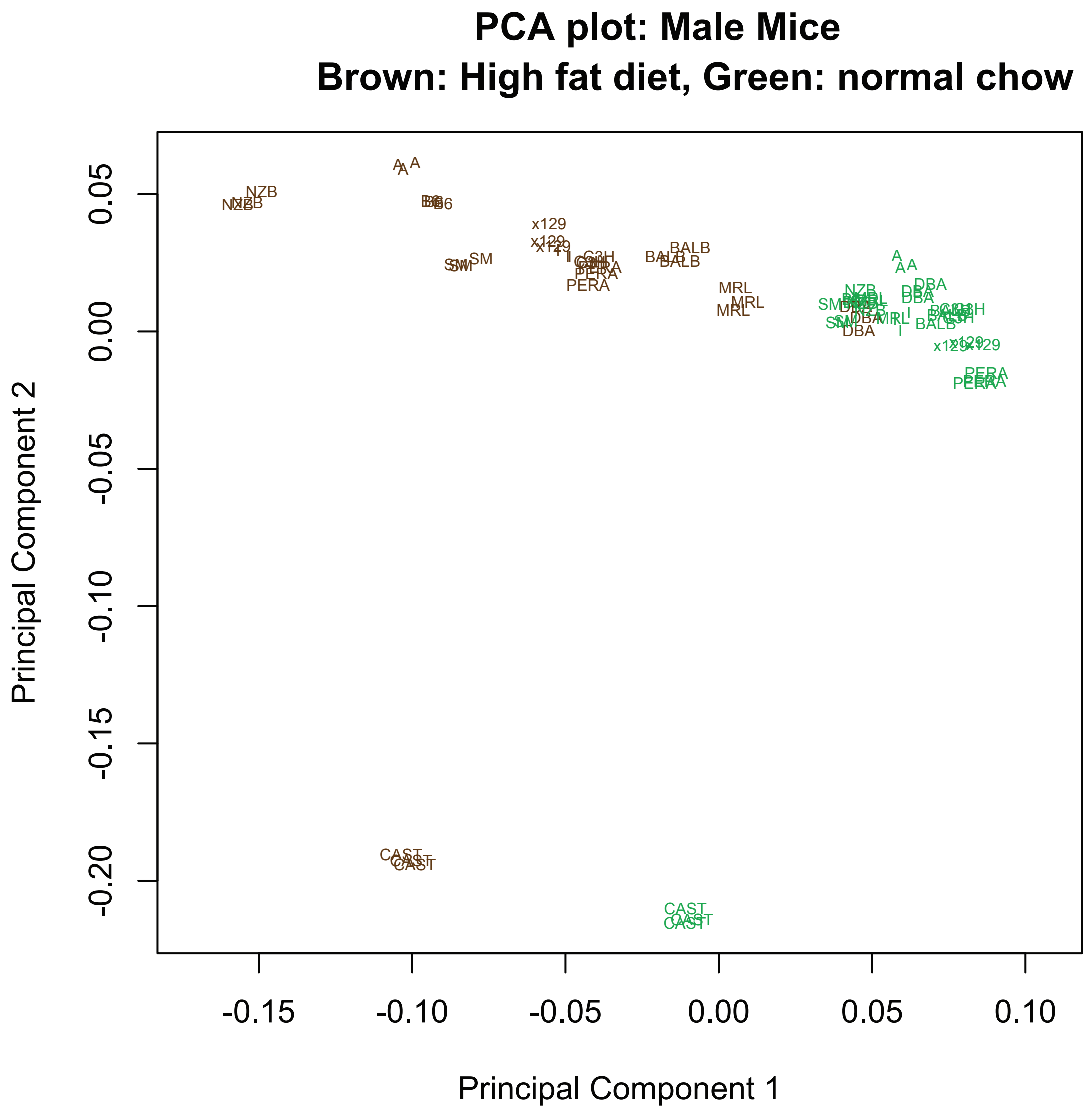

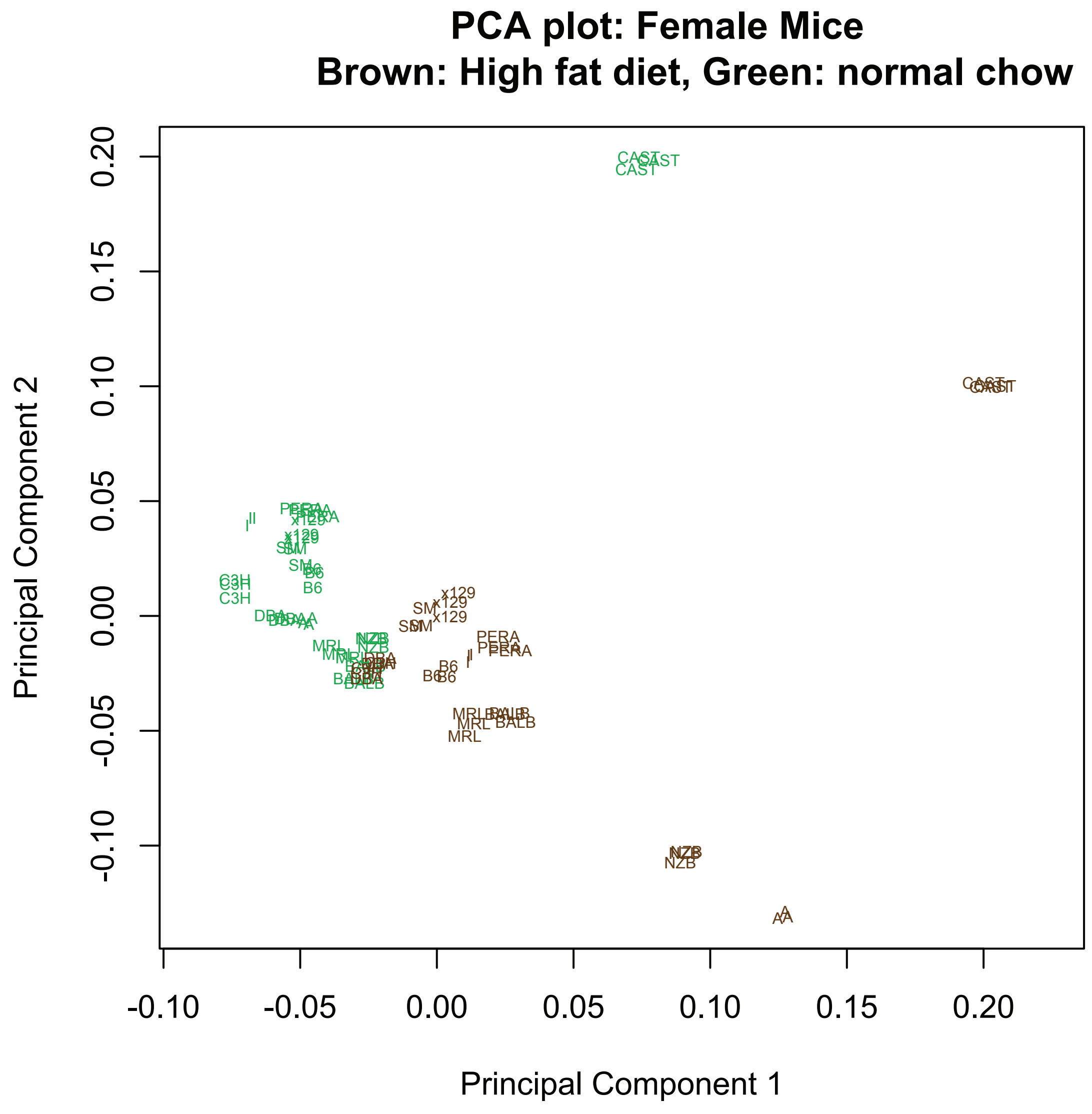

Suppl. Figure 1. Clustering and Principle Components Analysis (PCA) of transcriptome data shows high similarity between triplicate mice of a given strain-sex-diet combination, with variable influence of each factor depending on the specific comparison considered.
